# Advanced 3D Food Printing with Simultaneous Cooking and Generative AI Design

**DOI:** 10.1002/adma.202408282

**Published:** 2025-02-26

**Authors:** Connie Kong Wai Lee, Yang Xu, Qiaoyaxiao Yuan, Yee Him Chan, Wing Yan Poon, Haosong Zhong, Siyu Chen, Mitch Guijun Li

**Affiliations:** ^1^ Center for Smart Manufacturing Division of Integrative Systems and Design The Hong Kong University of Science and Technology Clear Water Bay, Kowloon Hong Kong SAR 999077 China; ^2^ Hong Kong Branch of Chinese National Engineering Research Center for Tissue Restoration and Reconstruction The Hong Kong University of Science and Technology Clear Water Bay, Kowloon Hong Kong SAR 999077 China; ^3^ Joint Laboratory for Wave Functional Materials Research The Hong Kong University of Science and Technology Clear Water Bay, Kowloon Hong Kong SAR 999077 China

**Keywords:** 3D food printing, additive manufacturing, generative AI, laser‐induced graphene

## Abstract

3D food printing is an indispensable technology for emerging food technologies. However, conventional nonconcurrent postprocessing methods limit the final food quality, including the unappealing nature of food ink modification, imperfections in retaining the desired food shape, and the risk of microbial contamination. Here, an artificial intelligence (AI)‐enhanced solution is developed to achieve extrusion‐based printing with simultaneous infrared heating, enabling in‐line and rapid cooking of complex starch‐based food. Noncontact graphene heaters as cooking sources present outstanding food quality control with microbial studies, microstructure analysis, and heat transfer simulation models. This integrative 3D food printing method with AI‐enhanced food pattern generation and in‐situ cooking significantly expands the applications for customized food creation. It paves the way for the broader adoption of heating‐based 3D printing of functional materials.

## Graphical abstract

An in‐situ heating solution for 3D food printing is developed by incorporating noncontact graphene heaters as cooking sources during the printing process. This technique ensures food quality control and reduces microbial contamination. The AI‐generated 3D food shape design allows users to create digital models using text input without prior computational skills.

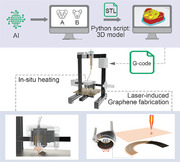

## 1 Introduction

3D printing technology enables food to be fabricated with consistency in shapes and ingredient compositions.^[^ ^1^, ^2^ ^]^ Compared with conventional food fabrication technologies, the advantages of 3D food printing include the ability to create customized food without the need for extensive product equipment modifications and reduced labor costs with its automated production process. With extrusion‐based 3D food printing, different types of food can be fabricated by modifying them into printable food ink.^[^ ^3^, ^4^ ^]^ Furthermore, it is necessary to postprocess certain food types by heating or drying.^[^ ^5^, ^6^ ^]^ Conventional postprocessing methods, such as oven baking and air‐frying, have limitations regarding retaining the desired food shape. Moreover, these postprocessing treatments must be carried out separately, which consumes more energy and time.^[^ ^7^ ^]^ On the other hand, microbial safety remains one of the challenges for 3D‐printed food, particularly in the context of in‐situ postprocessing and print‐to‐serve scenarios. Considerations such as deploying fabrication systems with reduced direct contact with food and shortening the food processing time can contribute to the final quality attributes of the 3D‐printed food.^[^ ^8^ ^]^ To overcome these limitations, in‐line cooking methods can help to retain the printed food's desired shape and eliminate the need for separate postprocessing steps.

In‐line heating methods during the food printing process have gained research interest recently, including preheating the printer's extruder, hot‐air heating, microwave‐assisted heating, and laser‐based cooking of the printed food during deposition.^[^ ^9^, ^10^, ^11^, ^12^ ^]^ While preheating the food ink before printing within the extruder system can eliminate the risk of microbial contamination, the final printed food is deposited at room temperature. It may not be desirable for consumption immediately. On the other hand, laser cooking has shown potential by increasing structural stability while delivering precision cooking or browning of specific areas of the food.^[^ ^6^, ^13^ ^]^ However, it can be inefficient due to the laser beam's limited ability to heat only a small area of food at a time, thus a long heating time.^[^ ^11^ ^]^ Furthermore, laser cooking systems require multiple complex components, including the laser lamp and galvo mirror systems, to achieve the desired cooking ability. Different laser beam sizes and power settings may affect the overall cooking performance. As the laser cooking system may pose a hazard to the retina, additional considerations in preventing the exposure of the laser beam must also be carefully examined. Conversely, infrared (IR) cooking is ideal for heating 3D‐printed food via thermal radiation. Recently, preliminary works on integrating IR and laser cooking systems with 3D food printers have been reported.^[^ ^14^, ^15^ ^]^ With IR cooking, heat is transferred through electromagnetic radiation, exhibiting advantages over traditional cooking methods, including rapid and uniform heating and higher thermal and energy efficiency. However, conventional IR cooking methods utilizing IR lamps have limitations regarding flexibility, energy efficiency, cost, and ease of integration. In contrast, advanced materials like laser‐induced graphene (LIG) offer advantages as cooking applications due to their high thermal emissivity, flexibility, and compact size.^[^ ^16^ ^]^ LIG has demonstrated its potential in various fields, such as energy storage, sensors, electronics, water purification, and biomedical applications.^[^ ^17^ ^]^ Its unique properties and versatility make it a promising material with untapped potential for treating food.

Herein, we propose an in‐line, simultaneous printing and cooking solution using a highly thermal emissive, flexible, compact, and integrated LIG IR heater. We fabricate the heater from a low‐cost polyimide (PI) film in a one‐step laser‐assisted manufacturing technique with high precision at the micro‐scale without manufacturing costly casting. While prior works have documented the laser ablation process of graphene heaters,^[^ ^16^, ^17^ ^]^ none were designed to apply in 3D food printing and food research. We integrate the heater device with the 3D printer nozzle unit to achieve a low‐cost, energy‐saving integrative solution for in‐line postprocessing 3D‐printed food. Furthermore, the integrative print and cook system reduce both manpower and equipment costs as there is no need to transfer and postprocess the printed food products with additional equipment. By utilizing the advantage of its flexibility and ultra‐thin thickness, we could design a predefined pattern and shape of the heater to optimize its thermal properties and generate a focused and targeted heating source. Hence, our research explores using advanced novel materials to enhance food processing techniques, revolutionizing 3D‐printed food postprocessing methods. We achieve real‐time printing and heating to increase the printability of starch‐based cookie dough. Our result shows an even heat distribution and effective heat transfer from the heater unit to the printed food layers.

In the 3D food printing preparation phase, 3D modeling programs create customized food shapes.^[^ ^1^ ^]^ While individuals with computational modeling skills can manage the process of 3D food printing, it can pose a barrier for those without these skills who want to create their customized designs. Artificial intelligence (AI) has made generating images based on text descriptions possible in recent years.^[^ ^18^ ^]^ On the other hand, Python, a versatile and widely used programming language, has gained popularity in Science‐Technology‐Engineering‐Mathematics (STEM) education, equipping individuals from a young age with the skills necessary to leverage AI tools and algorithms effectively. This has streamlined the development and implementation of AI‐driven solutions in various sectors, including the food industry, leading to increased efficiency, enhanced creativity, and accelerated innovation in food design, production, and customization. In this work, we utilize AI and Python to solve and overcome technical barriers to 3D food printing. We propose a streamlined method using text‐to‐image algorithms and image‐to‐mesh conversion calculations to aid the 3D food design process. We successfully printed 3D food shapes using simplified 2D images created by generative AI. By leveraging AI's capabilities and Python programming tools, the design process can incorporate user feedback in real time, allowing for iterative improvements that cater to individual tastes and needs, thus enhancing user satisfaction. This approach simplifies the design process, reducing the need for extensive computer graphics and design skills.

## 2 Result

**Figure 1:**
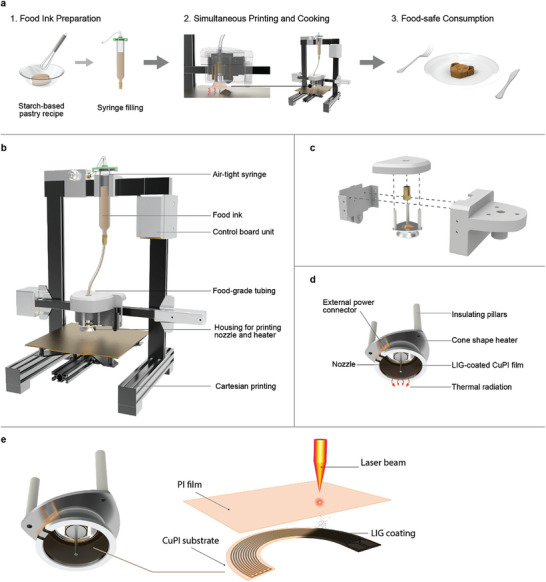
a). The step‐by‐step food 3D food fabrication process of the printing and in‐line cooking device. b). Design features of the integrative 3D food printer. c) The print head unit has an extrusion tubing inlet, extrusion nozzle, and heater holder. d) The external shell of the infrared heater has a cone‐shaped design to converge heat transmission to the targeted printing area. e) The schematic diagram of the fabrication of the LIG infrared heater.

**Figure 2:**
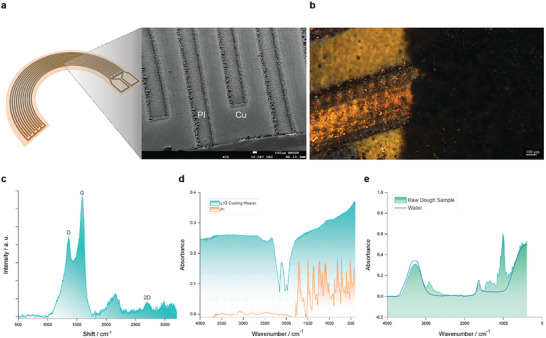
a) Schematic diagram and scanning electron microscopy image of the LIG heating film. b) Optical microscope images of the LIG heating film. c) Raman spectra of the LIG heating film. d) Fourier transform infrared spectroscopy of the LIG heating film. e) Absorption spectrum of the printed dough sample.

A device for printing and cooking in‐line is developed to maintain the desired shape (**Figure** **1**a). A complex starch‐based food ink is chosen to be the modeling food system. Starch is one of the primary carbohydrate diets and a widely used food thickening agent.^[^ ^19^ ^]^ According to previous studies, starchy products are considered as printable food ink due to their shear‐thinning behavior, allowing food ink to be extruded from a fine nozzle.^[^ ^20^ ^]^ However, starch products with wheat flour are processed with poor final printing quality due to the weak‐bodied nature of their printed paste. It is, therefore, essential to study the printability of wheat flour dough recipes as a desirable food ink. The proportion of water, wheat flour, oil, and water must be calculated to achieve a consistent viscosity suitable for printing. Among various extrusion mechanisms, air pressure‐driven extrusion is adapted to extrude food ink with high viscosity by adjusting the air pressure input via a connected air pump. Prepared food ink is transferred to an air‐tight syringe for food extrusion. In the air pressure‐driven extrusion, there is no direct contact between the mechanical components and food material, minimizing the risk of contamination. A regulator is integrated into the printing system to ensure consistent and steady air pressure throughout the printing process. An in‐line integrative heater is mounted on the print head unit to simultaneously print and cook the extruded food layers. Contactless cooking is achieved with radiative heat transfer from the cone‐shaped heater to the targeted food layers. This one‐step print‐and‐cook solution can deliver ready‐to‐eat food products, minimizing food fabrication and providing safe food consumption (Figure 1b; Figure S1, Supporting Information). The integrated print head unit is housed with the LIG heater and printing nozzle. Extruded food is heated once it is deposited onto the print bed. The Cartesian printing platform is used to minimize the movement of the print head unit for better printing resolution. Temperature‐controlled print beds aid in solidifying the base layers of printed food. The print head unit comprises an extrusion tubing inlet, an extrusion nozzle, and a heater holder (Figure 1c). Specifically, the external shell of the IR heater is made of aluminum to provide rigidity and stability to the shape. The cone‐shaped heater is designed to converge heat transmission to the targeted printing area (Figure 1d). The overall mechanism, including the print head, extrusion tubing inlet, extrusion nozzle, and heater holder, was incorporated and housed in a covered compartment to ensure the safety and cleanliness (Figure 1e). The ultrathin precut copper‐cladded polyimide (CuPI) film is a precisely cut U‐shape that can be bent after processing and fit into the inner wall of the cone‐shaped aluminum mount (Figure S2, Supporting Information). Non‐uniformity in temperature is a common problem for planar heating elements due to the difference in surface current density. To tackle this problem, we created a predefined pattern of copper wires that enhances heating uniformity through well‐spaced copper conductors. This optimized patterned serpentine circuit was laser scribed on the CuPI film, ensuring an evenly distributed flow of current across a large surface (Figure S3, Supporting Information). This method enables the fabricated LIG‐coated PI film to be the heat‐uniforming layer, achieving a uniform temperature distribution. This process was created in a one‐step laser induction and deposition process via the laser's high temperature at the focal point. It converts the PI layer into LIG, which serves as the blackbody thermal radiation source. Furthermore, LIG‐coated materials process better emissivity with acceleration in heat transfer.^[^ ^21^ ^]^ In our work, LIG on the CuPI heating film is deposited via a laser‐induced transfer method. LIG coating with good adhesion is achieved within its compact form factor at a dimension of 35 mm radius and 10 mm width (11 cm^2^ area). To study the morphology of the fabricated heating film, images were taken from both a scanning electron microscope (SEM) and an optical microscope (OM) (**Figure** **2**a,b). The OM image illustrates the successful laser ablation of the predefined copper pattern on the PI film. The SEM image illustrates the surface details of the laser‐patterned film with PI after ablation and pristine copper parts. Raman spectral analysis was conducted to confirm the successful laser‐induced transfer of graphene onto the CuPI film (Figure 2c).

To understand the correlation between the LIG IR heating element and the printed dough, Fourier transform infrared (FTIR) spectroscopy studies were conducted on the LIG heating element and printed dough, respectively. The characteristic peaks correspond to the unique absorption spectrum, which characterize the emissivity of the LIG heating material (Figure 2d). The absorption spectrum of the printed dough sample reveals distinct infrared absorption bands (Figure 2e). We confirmed that the  emission spectrum of the IR heating aligns with the printed dough's absorption peaks, indicating an effective heat transfer process.

**Figure 3:**
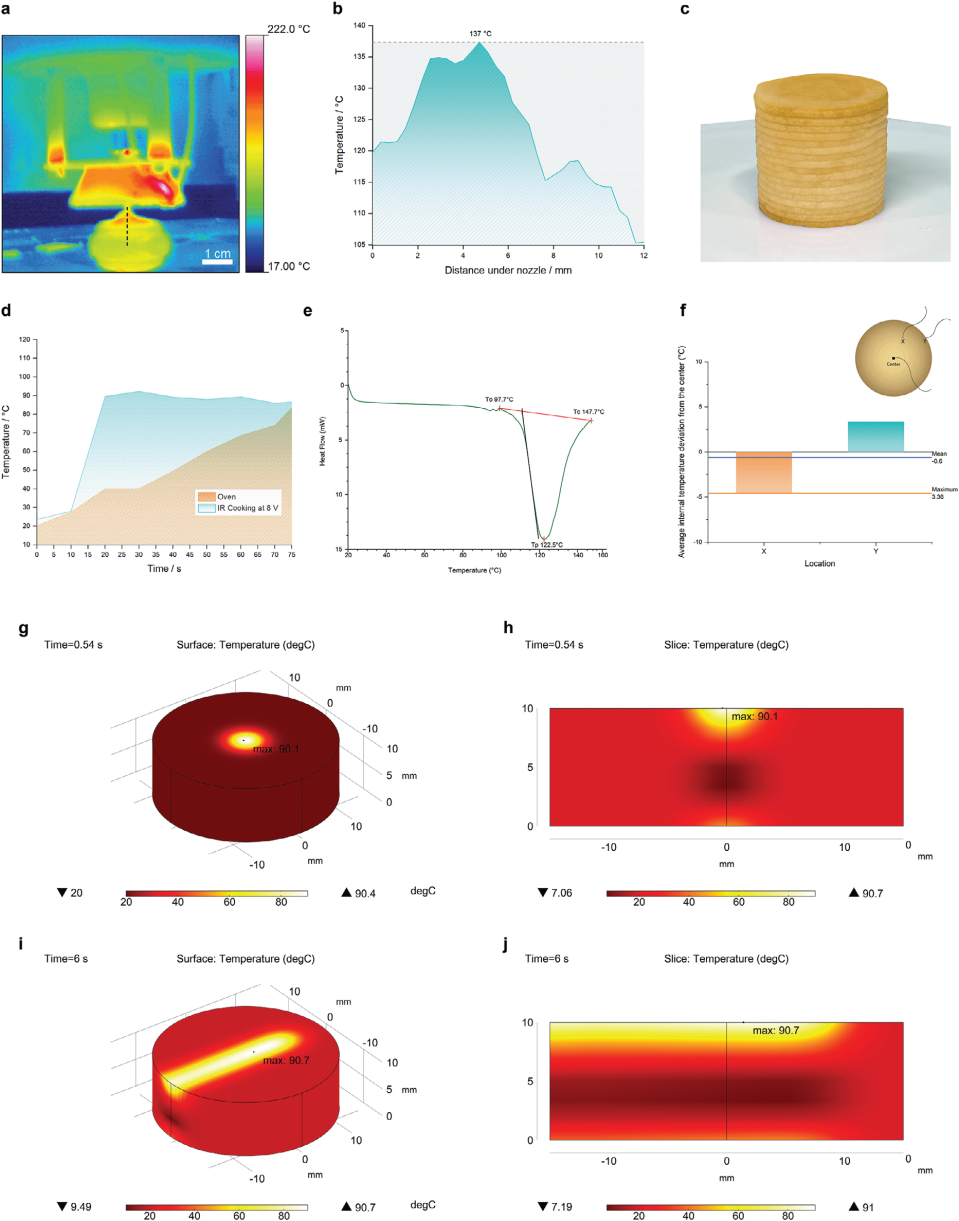
Temperature profile of the printed dough. a) An IR camera image of the printed dough. b) The internal temperature of the printed dough during the printing process. c) Illustration figure of the printed dough sample treated with infrared cooking. d) Temperature profile of LIG infrared heater and conventional oven baking. e) Differential scanning calorimetry graph of the raw dough sample. f) Average internal temperature deviation from the center of the dough sample during printing. g) COMSOL simulation of the infrared cooking on the 3D‐printed dough sample. h) Cross‐section view COMSOL simulation of the infrared cooking on the 3D printed dough sample. i) COMSOL simulation of the movement of infrared cooking on the 3D‐printed dough sample along the *x*‐axis for 6 s. j) Cross‐section view COMSOL simulation of the movement of infrared cooking on the 3D‐printed dough sample along the *x*‐axis for 6 s.

The IR camera imaging and internal temperature profile of the printed dough demonstrated that the integrated IR heater could provide penetrative heating to the printed dough (**Figure** **3**a–c). At a constant power output of 8 V, the surface temperature of the printed dough reaches 137 °C, and its side temperature maintains above 105 °C throughout the printing process. The flat‐surface heater's heating efficiency is much lower, reaching only a maximum surface temperature of 64 °C on the printed dough (Figure S4, Supporting Information). Therefore, the cone‐shaped heater can converge heat transmission to the targeted heating areas. On the other hand, while the energy‐saving LIG heater only consumes 14 W to operate, the oven and air‐fryer consume at least 1000–2000 W of power to achieve a similar cooking effect. The heating efficiency of the LIG IR heater is further compared with conventional oven baking (Figure 3d). The LIG IR heater reaches an initial temperature of at least 89.5 °C at the 20‐s interval, while the conventional oven takes more than 1 min to reach a similar temperature. The shorter amount of time needed to reach the targeted temperature ensures consistency in temperature uniformity throughout the cooking process. Furthermore, the IR cooker has a much higher energy‐saving efficiency than conventional baking methods.

On the other hand, the oven baking method reveals the risk of temperature overshooting. In this control experiment, the oven‐baked food samples exhibited a discrepancy between the internal temperature and the desired temperature setting in the oven, emphasizing the importance of implementing a software‐controlled cooking method for precise cooking. Moreover, the postprocessing step of baking 3D‐printed food with domestic ovens can result in food deformation, leading to undesirable outcomes such as food shrinkage and deformed food samples.^[^ ^22^, ^23^, ^24^, ^25^ ^]^ These undesirable outcomes can negatively impact the taste and texture of the final food product, ultimately reducing its quality and consumer satisfaction. Additionally, the low and slow increase in the initial temperature encountered in oven baking further emphasizes the need for an alternative cooking approach to deliver more precise and rapid heating.

The differential scanning calorimetry (DSC) graph shows that the onset gelatinization temperature is observed at 97.7 °C, while the peak temperature is at 122.5 °C (Figure 3e). While starch generally gelatinizes at ≈70 °C, the resulting higher gelatinization temperature of the raw dough may be due to the presence of sugars and oils.^[^ ^26^, ^27^ ^]^ The DSC result confirms an effective thermal treatment of the dough with our postprocessing method by cooking at a temperature higher than the gelatinization temperature of the dough. The power output of the IR heater is adjusted in terms of voltage and current values to match the dough's gelatinization temperature range accordingly.

To evaluate the heating distribution and uniformity of the printed food, the average internal temperature deviation from the center temperature was analyzed during the printing process for 30 min (Figure 3f). The maximum deviation is 3.36 °C at location x, while the mean deviation is 0.6 °C. This result ensures the uniform cooking of the printing food within a range of ± 4 °C, with an average of less than 1 °C in different food locations.

Furthermore, we use COMSOL simulations to study the heat transfer process and spatial temperature profile of the printed dough (Figure 3g–j; Video S1, Supporting Information). Material properties of each ingredient, including thermal conductivity, heat capacity, and density, are identified and calculated based on the recipe formulation with weighted averages, respectively.^[^ ^28^, ^29^ ^]^ Simulated heat flow is applied at 6.5 W onto the dough, distributed in a Gaussian beam with a radius of 4 mm (Figure 3g,h). Gaussian heat flux is used to simulate the central‐peaked heat flux in accordance with the experimentally measured results. The movement of the heat flow is simulated along the *x*‐axis for 6 s to simulate the printing head movement at a speed of 4.5 mm s^−1^ (Figure 3i). The cross‐section view of the IR‐cooked dough model along the xz plane shows that the heat penetration is between 1 and 2 mm measured from the top layer of the printed dough (Figure 3j). This simulation model illustrates the heat transfer and radiative cooking process only affects the top printing layer without overcooking the printed dough layers underneath, which is ideal for 3D food printing with layer‐to‐layer fabrication for targeted and focused heating. Furthermore, factors including thermal conductivity, heat capacity, and density of each ingredient can affect the efficacy of the heat transfer process. For instance, food with high moisture content tends to have a lower heating temperature, as shown in our COMSOL simulations (Figure S5, Supporting Information). Based on the simulation results, the optimized heat flux power is adjusted to provide an adequate heat penetration depth according to different food types and printing parameters, such as nozzle size and layer height.

**Figure 4:**
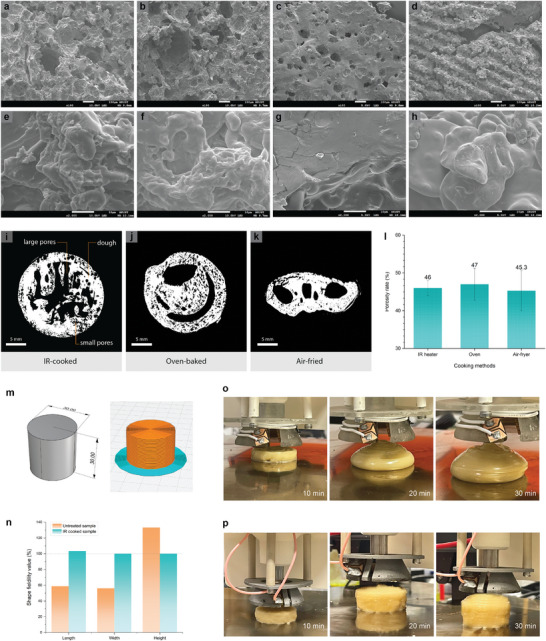
a–d) SEM images at ×100 magnification of starch‐based dough samples processed with air‐frying, oven‐baking, IR cooking, and laser cooking (from left to right). e–h) SEM images at ×2000 magnification of starch‐based dough samples processed with air‐frying, oven‐baking, IR cooking, and laser cooking (from left to right). i–k) Reconstructed micro‐computed tomography 2D cross‐section images of the printed dough with different cooking methods. l) Porosity rate (%) of the printed dough with different cooking methods. m) Printability test using a cylindric shape of 30 × 30 × 30 mm as the testing model. n) Shape fidelity value (%) of untreated and IR‐cooked samples in terms of length, width, and height. o) 3D printing process of the starch‐based dough without heat treatment for 30 min. p) 3D printing process of the starch‐based dough with LIG treatment for 30 min.

SEM was conducted to compare the effect of different cooking methods on the microstructural behaviors of the cookie dough. (**Figure** **4**a–h). At ×100 magnification (Figure 4a–d), cookie dough samples with air‐frying, oven‐baking, and IR cooking treatments (Figure 4a–c) show visible porous structures. SEM images can further be used to understand the gelatinization process as intact starch granules in raw uncooked cookies will be lost upon gelatinization.^[^ ^30^ ^]^ At ×2000 magnification (Figure 4e–h), all examples, except for the laser‐cooked sample, show a network of granular structure without the presence of intact starch granules, indicating the gelatinization process has taken place. Conversely, the starch granules of the laser‐cooked sample are still intact. Moreover, the granular structure of the IR‐cooked sample appears to be less swollen than the air‐fried and oven‐baked samples, which corresponds to the macrostructure of the dough with a more retained shape. This behavior can be explained by the quick and even IR heating that may promote a more homogeneous structure in the starch matrix with its moisture retention characteristics. Similar behaviors and observations of cooked gluten‐starch structures with higher water‐holding capacity showing a dense and compact starch granule network were reported.^[^ ^31^ ^]^ On the other hand, oven‐baking and air‐frying involve indirect heat transfer through convection and conduction. The heating process causes dramatic starch welling within the food, potentially leading to the formation of a more dramatic size increase of the starch granules with deformed shapes.^[^ ^32^ ^]^ Furthermore, the laser‐cooked dough sample showed traces of obvious laser cooking paths with a great degree of surface roughness (Figure 4d). It was observed that the starch‐gluten matrix was not yet fully hydrated, indicated by intact starch granules without forming the gluten network (Figure 4h). This observation suggests that laser cooking can result in producing a rough surface texture, and the gaps between each laser cooking beam may hinder the food from being fully cooked.

To further characterize the internal structural properties of the food with IR cooking, we used the X‐ray micro‐computed tomography (micro‐CT) method to visualize and quantify the internal porosity structures, which contribute to the overall texture properties.^[^ ^31^, ^33^, ^34^ ^]^ In this case, porosity refers to the ratio of the small pores in the dough to its total volume. 2D images were reconstructed from the 3D model of the scanned dough (Figure 4i–k; Figure S6, Supporting Information). The internal structure of the IR‐cooked dough is well retained compared to the other two samples, where the larger pores resemble the infill pattern of the dough. The infill patterns in oven‐baked and air‐fried samples are less obvious due to the deformation in shape during the postprocessing methods. To maintain accuracy in our porosity calculation, the large pores are excluded from our analysis. The cross‐section images reveal that the IR‐cooked dough exhibits an average porosity rate of 46% (Figure 4l). On the other hand, the oven‐baked dough shows a porosity rate of 47%, while the air‐fried dough demonstrates a porosity rate of 45.3%. The porous structure can be explained by the expansion and breakage of air bubbles during the cooking process.^[^ ^34^ ^]^ Although there are noticeable deformations in the oven‐baked and air‐fried samples due to poor shape retention during the printing and postprocessing treatments, the porosity levels of all the samples are comparable. The results of the micro‐CT images align with the SEM images, where the IR‐cooked dough undergoes gelatinization while retaining its structural integrity.

The printability evaluation study used a cylindric shape of 30 × 30 × 30 mm as the testing model (Figure 4m). The cylindric shape was printed for 30 min to examine any deformation issues under a long‐term printing process. Printing parameters such as infill rate, pattern, nozzle size, printing speed, and layer height were adjusted to achieve an optimal printing result. The cookie dough was printed with a dense infill closely resembling conventional cookies to enhance overall consumer acceptance. The shape fidelity of untreated and IR‐cooked samples was analyzed (Figure 4n). No cracking or collapsing was observed at any part of the printed cylinder. Shape fidelity values within the 100 ± 5% range are considered optimum retention.^[^ ^35^ ^]^ While the intended cylindric shape was 30 × 30 × 30 mm by design, the measured dimension of the extruded untreated sample at the 30‐min interval was 51 × 53 × 15 mm, resulting in an average of 82.7% shape fidelity value. Significant deformation and shape deviation occurred with the untreated sample at the 20‐min interval (Figure 4o). With LIG IR heating, the shape retention value is 103% at the 30‐min interval (Figure 4n,o; Video S2, Supporting Information). Printing tests of more complex shapes including shapes with perforated patterns, a 3D boat model, and a standing owl model were further conducted to study the shape retention capability (Figure S7a–d, Supporting Information). The resulting printed 3D shapes with perforated patterns resemble the intended designs. Both the 3D toy boat model with a raised bow structure and the standing owl model demonstrated good shape retention during the printing process without collapsing or severe deformation. This study further verifies that our heater aids in preserving the shape and structural stability of the printed dough throughout the 3D printing process. Furthermore, printing tests using various raw ingredients such as vegetable puree and chicken puree are successfully printed with good shape retention (Figure S7e, Supporting Information), showcasing the potential of applying the IR cooking method to various kinds of food categories.

### 2.1 Safe Food Consumption

**Figure 5:**
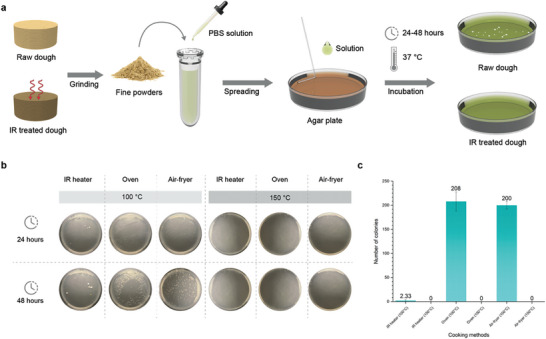
Microbial studies of the printed dough. a) Schematic diagram of the bacterial testing procedures to evaluate the efficiency of the LIG infrared heater. b) Colony counting images of the printed dough with different cooking methods at 100 and 150 °C post cultivation. c) Number of colonies of the printed dough with different cooking methods at both 100 and 150 °C.

To evaluate the safety aspect of our in‐line cooked food samples, total viable count (TVC) bacterial tests were conducted with IR‐cooked, oven‐baked, and air‐fried samples at both 100 and 150 °C to compare the bacterial inhibition efficacy (**Figure** **5**). In raw uncooked samples, widespread clusters of colonies were evident across the entire plate after 24 h, with continued growth at the 48‐h interval (Figure S8, Supporting Information). Compared to oven‐baked and air‐fried samples, IR‐cooked samples exhibited the lowest colony counts, ranging from 0 to 6 colonies after 24 h. By the 48‐h interval, colony numbers remained consistent while their sizes increased from small (<1 mm) to medium (1mm) and large (>1 mm). On the other hand, the samples prepared using oven‐baking and air‐frying exhibited significantly higher plate counts, exceeding 200 colonies at both the 24‐h and 48‐h intervals. The sizes of these colonies grew into medium‐sized and large‐sized colonies at the 48‐h interval. In conventional scenarios, cookies are typically oven‐baked with at least 150 to 175 °C. Therefore, the presence of bacterial growth in the samples cooked at 100 °C could be due to the need for longer cooking durations and a higher temperature to achieve microbial safety. The least amount of colony counts with IR‐cooked samples at 100 °C indicates that our in‐line cooking process effectively minimizes bacterial growth compared to conventional methods at equivalent temperatures. This result can be attributed to the layer‐by‐layer print and cook system that provides even and consistent penetrative thermal treatment to the printed food. On the other hand, all samples being cooked at 150 °C exhibited no colony count. The high heat at 150 °C likely played a significant role in effectively inhibiting bacterial growth, regardless of the cooking method used.

### 2.2 Artificial Intelligence (AI) Design Process Optimization

**Figure 6:**
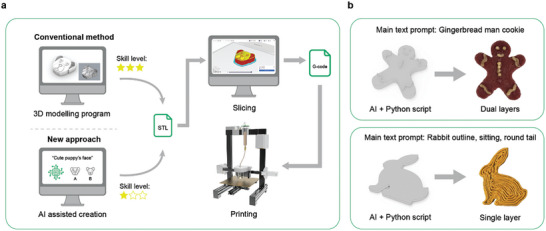
a) Food shape design process and workflow using AI diffusion models and Python scripts, compared to the conventional preparation method. b) Final 3D‐printed food samples based on generated 3D files utilizing AI text prompts and Python script.

Food shape design process and workflow using the AI diffusion model and Python scripts are proposed (**Figure** **6**a). Although some 3D food printer companies offer a limited preset library of food shapes, individual user customization is impossible.^[^ ^3^, ^36^, ^37^ ^]^ To tackle this problem, a method for utilizing AI to generate printable and unique patterns for 3D food extrusion printing on cookie dough was developed. Simplified 2D images were generated to convert into extruded 3D shapes via Python code. The generated designs are implementable with the chosen food material without further ingredient composition alterations.

Two vector graphic generation exercises were conducted using DALL‐E's optimized AI generation workflow, including a gingerbread man and a rabbit. Using the customized Python scripts, the downloaded 2D images are converted into STL 3D modeling files, which can be used for 3D printing. Users can easily run the script without coding knowledge by inputting the prompt with the desired extrusion height (Figure S9, Supporting Information). The generated STL files from diffusion models can be directly imported into open‐source printer slicing programs to generate gcode files. This easy‐to‐use workflow eliminates the need for 3D modeling software, shortening the 3D food design process. Both dual‐layered and single‐layered 3D models are successfully fabricated according to the generated AI image and Python script (Figure 6b). The script extrudes the secondary color with a height that is twice the height of the base layer. An in‐line IR cooking method was introduced to improve printability and shape retention. The results obtained are highly identical to the original 2D image, validating the accuracy using mathematical models. There are no signs of missing parts in either the generated 3D file or the final printed samples.

## 3 Discussion

The in‐line IR cooking method has proven effective in solidifying extruded food layers without alternating the food composition. It heats the printed food through radiative thermal heating during the printing process to improve structural stability, which is a common challenge with conventional postprocessing cooking methods. Aside from starch as the main ingredient in preparing cookie dough food ink for 3D food printing, additional thickening agents such as gums are added as modified food ink to enhance printability, flow properties, and stability. However, these agents, including Xanthan Gum and Gua Gum, may alter the flavor of the food.^[^ ^38^ ^]^ In contrast, the in‐line IR cooking method as a thermal stabilization aid is preferred as it prevents shape deviations, preserves taste, and maintains portion sizes. The intense and localized heat generated by the IR heater facilitates the rapid formation of a heat‐affected zone, which helps bond and solidify the layers of the extruded food. This promotes better shape retention and structural stability, reducing the likelihood of deformation and collapse. Therefore, the in‐line IR cooking method offers an efficient solution when compared to postprocessing challenges in 3D food printing, particularly with food types that are commonly baked or air‐fried. The one‐step fabrication of the LIG heater via a laser‐assisted manufacturing technique offers the advantages of cost‐effectiveness, design flexibility, and scalability. Furthermore, it is crucial to confirm that the absorption peaks of the food ink align with the emission spectrum of the IR heating source to ensure an effective heat transfer process. While IR cooking can inhibit bacterial growth effectively, it can also maintain the moisture content of food, which is an important factor in enhancing texture and taste.^[^ ^39^ ^]^ Furthermore, IR cooking is characterized by its rapid heating capabilities, resulting in a shorter cooking time than conventional cooking methods. This rapid cooking process can prevent the breakdown of flavors and textures and preserve food's nutritional quality and sensory properties.^[^ ^40^ ^]^ IR cooking also allows for the development of Maillard reactions, a desirable browning effect of cooked foods, including pastry items such as baked cookies and meat. Observed from our experiment results and previously reported works,^[^ ^41^ ^]^ IR heating may reveal differences in the overall internal structure of printed food compared with conventional methods, which can introduce different textural and sensory properties such as hardness and cohesiveness. Our research on the IR heating system enables precise targeting of specific layers or areas of the food during the printing process. This capability distinguishes our technology from traditional postprocessing methods, such as baking or air frying, which cannot be performed in‐situ alongside printing. Adjustments and alterations within the food item during printing are not feasible with these conventional methods. Moreover, heat transfer simulation models can help predict and provide insights into the heat transfer efficacy based on each food ingredient's material properties. Furthermore, a comprehensive understanding of the raw food ingredient's food‐safe temperature for consumption is crucial in ensuring that the cooking process is effective and safe. The proposed in‐line IR cooking method offers several advantages over conventional laser cooking systems, including a larger and evenly heating area, smoother surface texture, a reduced cooking time, and a compact and low‐cost setup. It comprises a more accessible and safer heating system that can be integrated with 3D food printing platforms.

Our study reveals that IR cooking presents a significant advantage in maintaining the shape and form of 3D food designs, enhancing visual appeal for consumers compared to traditional postprocessing methods (Figure 4m–p). In the realm of 3D‐printed food, user preferences, including shape, taste, and fabrication fidelity are considered important factors.^[^ ^42^ ^]^ We conducted a small blind taste test with four individuals, all of whom preferred the IR‐cooked food sample. They noted that while they found the texture and taste to be similar to the other samples, the food's appearance was more attractive. Our testers are satisfied with the printed food's appearance, which is highly accurate and closely resembles the intended design. To validate the texture of the IR‐cooked dough, we conducted a hardness analysis with a pin penetration test (Figure S11, Supporting Information). Hardness is a critical factor in evaluating the texture of cookies cooked by different methods such as IR cooking, oven baking, and air frying due to its direct correlation with perceived quality and consumer satisfaction. While each cooking method presents a slightly different force‐loading curve, the results show that there is no significant difference in the hardness level among the IR‐cooked, oven‐baked, and air‐fried samples. The results show that there is no significant difference in the hardness level among the IR‐cooked, oven‐baked, and air‐fried samples. Moreover, due to the unique advantage of heating and cooking the printed food layer‐by‐layer, adjustable heat flux power can help to introduce various textural properties within one food item. This can further enhance the final food quality and sophistication of the printed food in terms of introducing various mouthfeels and textures when compared to conventional postprocessing cooking methods like baking. Moreover, the color and browning of dough products is an important indicator of good quality. In our study, IR‐cooked dough reveals browning upon treatment and is considered desirable by our testers (Figure S12, Supporting Information). The effective utilization rate of the food ingredients being used in our work is also relatively higher compared to conventional food printing systems. With our in‐situ heating device, we are able to increase the success rate of printing the food product with high accuracy without food deformation. The rapid cooking process ensures that the printed food is safe for consumption, hence reducing the need to dispose of raw printed food that has been exposed to air for a long time.

Conventionally, in the first step of a 3D design process, the desired shape outline needs to be drawn manually as extrusion paths. The drawn paths should be extruded manually using computer graphic software programs like Rhino and SolidWorks, which require extra effort. Computer 3D visualization is a complex process requiring in‐depth knowledge.^[^ ^43^ ^]^ On the other hand, generative AI can help streamline the design process and stimulate creative thinking.^[^ ^44^ ^]^ Furthermore, fundamental programming skillsets using Python have been widely adopted in early childhood STEM‐based education.^[^ ^45^ ^]^ While common types of AI tools like ChatGPT have gained popularity in various applications like natural language processing and conversation generation,^[^ ^46^ ^]^ the use of image‐generative AI models, including Stable Diffusion, DALL‐E, and Mid‐journey is still emerging and holds significant potential for revolutionizing the field of visual content generation and artistic expression. Various techniques, such as text‐to‐mesh modeling, have been developed to translate textual descriptions into 3D shapes.^[^ ^47^ ^]^ However, these generated 3D models are unsuitable for 3D food printing due to the complex shapes and overhanging structures. On the other hand, the AI design generation methodology described in this study enables users to define specific characteristics and aesthetics to deliver printable extrusion paths with the simplified 2‐step procedures. Our AI‐enhanced approach revolutionizes food design and eliminates the need for manual drawing, streamlining the process. Python's accessibility empowers individuals without extensive computer graphics expertise to engage effectively, emphasizing creativity over technical skills and reducing the learning curve associated with complex 3D modeling software. By integrating text‐to‐image algorithms, the complexity of 3D food design is simplified. The approach of utilizing simple Python coding enables the direct conversion of AI‐generated images into 3D models without computer aided design software or advanced skills. This saves time and eliminates the need to navigate various 3D modeling software. Additionally, our custom script enables the creation of dual‐layered models from 2D images, enhancing design intricacy and accuracy preslicing. The close resemblance of the final results to the original image boosts user satisfaction and confidence, facilitating a more efficient and accessible design process for individuals at all skill levels. In optimizing the AI design process for creating unique 3D food printing models, ensuring that the generated designs reflect the user's tastes and preferences is essential. In our proposed AI‐assisted approach, users can provide specific text prompts to guide the AI model in crafting designs that align with their desired characteristics. Furthermore, enabling users to select from various generated images lets them pick designs that match their preferences visually. Implementing criteria and filters in platforms like DALL‐E can enhance personalization by allowing users to specify factors such as shape details. To avoid generating over‐complex shapes and structures that may not be suitable for food printing, a set of criteria was predefined in the AI model to prioritize simplified vector‐like images, outlines, or duo‐color formats for dual food material printing designs. By employing dynamic image generation and leveraging text‐to‐image algorithms, each design is guaranteed to be unique, effectively facilitating the creation of personalized 3D food printing models tailored to individual preferences. This level of customization and control over the 3D food design process can significantly enhance creativity and precision in food shape generation while ensuring the printability of the design. Our AI‐driven design process streamlines the generation of unique food designs efficiently and rapidly, catering to individualized customer needs, making this 3D printing design versatile for deployment in various settings such as restaurants, bakery shops, and other food service environments that demand a high degree of customization.

## 4 Conclusion

We developed a rapid, programmable, and integrative 3D food printing solution with an in‐line IR cooking method and an AI‐assisted design process. The proposed generative AI and Python scripts successfully printed 3D food designs. The shape retention studies, bacterial tests, microstructure analysis, and heat transfer simulations indicate the cooking efficiency of the LIG IR heater on 3D‐printed starch‐based food in terms of printing accuracy, shape retention, and final food quality. Moreover, in our blind taste test, all testers are satisfied with the food's appearance, fidelity, and taste of the printed food products.

Further investigations can be conducted to assess the impact of the LIG IR heating on preserving essential micronutrients, such as heat‐sensitive vitamins, and starch digestibility of the printed food. A combination of LIG IR heating and other cooking techniques, such as hot‐air heating and microwave‐assisted heating, can be studied to improve the final printed food quality, especially for more complex food shapes. Moreover, it would be valuable to investigate how the in‐line IR cooking method can aid the 3D printing process of other food categories, like protein‐rich food products, including temperature distribution and sensory properties. Specific pathogenic bacterial inhibition studies such as *E. coli* can be further evaluated. To ensure user‐friendliness and broader consumer acceptance of the proposed streamlined 3D food design workflow and IR‐cooked food products, future works could include sensory and consumer acceptance evaluations with target users, such as children or caretakers in hospitals. Multi‐nozzle 3D food printing can be utilized to extrude more complex 3D food shapes with fine details using secondary food ingredients as supporting materials. Extended studies can be conducted on how the AI‐assisted design process can aid the 3D printing of different food types and structural complexity that may have specific limitations. By involving these users in the evaluation process, researchers can gain valuable insights into the usability and practicality of the approach. In our future studies, we will focus on exploring the mentioned areas to enhance the overall consumer experience and ensure the successful integration of this innovative technology in the culinary domain. Ongoing challenges include understanding the diverse thermal properties and sensory preferences of different food types, as well as addressing the need for multi‐material capabilities and increased printing speed in a scaled‐up production setting. To overcome these challenges, the development of multi‐nozzle 3D food printing and cooking to create integrated nutritious meals for health management can potentially revolutionize nutritional care in extended application settings such as central kitchens in hospitals or care facilities.

## 5 Experimental Section

### Food Ink Materials

A starch‐based cookie dough comprises soft wheat flour, vegetable oil, sugar syrup, and water (Table S1, Supporting Information). All ingredients were purchased from a local supermarket.

### Preparation of Cookie Dough

To prepare the cookie dough for printing, sugar syrup, water, and vegetable oil were mixed in a large mixing bowl until well incorporated. Soft wheat flour was added to the mixture, and all the ingredients were mixed for 5 min until a uniform texture was obtained. The mixture was covered with plastic wrap and rested for 30 min for consistent texture and elasticity. The rested cookie dough was then loaded into the printer's syringe of 50 mL for 3D printing.

### 3D Printer Design and Development

We built our customized food printer using a commercially available filament extrusion‐based Anycubic Kobra (Table S2, Supporting Information). We replaced the extrusion mechanism with an air pressure‐driven system for food extrusion using food‐grade syringes and polyurethane tubing. We mounted our proprietary integrated IR heater to simultaneously cook the extruded food layers. An electronic platform with Arduino systems activated and controlled corresponding compartments and systems.

### Laser‐induced Graphene (LIG) Coated Heating Film and Heater Mount

A laser source of a 1064 nm pulsed laser marking machine was used to laser scribe the predefined patterning on the copper‐cladded polyimide film with a thickness of 35 µm copper and 17 µm PI. The CuPI film was coated with a thin layer of LIG from a 50 µm thick PI film via the 1064 nm pulsed laser. The fabricated LIG‐coated heating film was then assembled and fixed on the inner wall of the heater's holder.

### Characterization and Measurement

SEM images were captured to study the surface details of the LIG‐coated heating film. FTIR spectroscopy and Raman spectral analysis were conducted to study the absorption spectrum and the presence of LIG. IR imaging and K‐type thermocouples were used to obtain the temperature profiles of the printed dough (Figure S10, Supporting Information). FTIR absorption spectroscopy was used to study the absorption spectrum of the LIG and the dough. DSC (Model Q1000 Calorimeter, TA Instruments) was used to observe the thermal properties of the starch‐based cookie dough. 4 mg of raw cookie dough samples were scanned from 20 to 150 °C at a heating rate of 10 °C min^−1^. SEM was conducted to study the microstructural behaviors of the food samples with different postprocessing methods, including air‐frying, oven‐baked, IR cooking, and laser cooking (Figure S12, Supporting Information). The prepared samples were cut and coated with gold using a gold sputter coater (Model K575xd, Emitech Ltd.) for conductivity enhancement. Images were obtained using SEM (JEOL‐7100F), and different image magnifications were taken in sequence.

The internal structures of the printed dough with different cooking methods were characterized using X‐ray micro‐CT, operating at 100 kV and 67.0 µA. The sample was placed on a sample holder on a rotating stage for scanning. 1800 projections were captured with a scanning angle of 360° to obtain the reconstructed cross‐section images at ×3.8 magnification.

The shape retention test used a cylinder shape with a 30 × 30 × 30 mm dimension as a control model. The 3D model was prepared in Rhinoceros and was sliced into a gcode file in Cura, an open‐source slicing program for 3D printing. Photos of extruded food were taken during different time intervals, and a caliper was used to measure the dimensional characteristics of the printed samples. Shape fidelity rate (Equation 1) was adapted to study the deformation of each printed sample:^[^ ^35^ ^]^

(1) $$\begin{array}{ccc} \mathrm{Shape}\;\mathrm{fidelity}\left(\%\right) & = & \mathrm{Measure}\;\mathrm{dimension}\left(\mathrm{mm}\right)\\ & & ÷\mathrm{Theoretical}\;\mathrm{dimension}\left(\mathrm{mm}\right)\times 100 \end{array}$$

The pin penetration test was used to measure the force‐loading curves of IR‐cooked, oven‐baked, and air‐fried cookie dough samples (Figure S11, Supporting Information). A motorized positioning system (PDV PP110‐30‐5040) was used to drive a pin with a 2 mm diameter for the penetration tests.

### Bacteria Testing for Safe Food Consumption

Using different cooking methods, bacteria were tested using the TVC method to assess bacteria growth in printed food. Printed dough samples were ground into fine powders. The 0.1 g of the powder was dissolved with 500 µL of phosphate‐buffered saline solution before being spread onto Petri dishes containing agar plates for incubation. The Petri dishes were cultivated at 37 ^○^C for up to 48 h to detect the growth of any potentially present bacteria.

### Generative AI Model

DALL‐E, an AI‐powered deep learning text‐to‐image generative neural network, was used to generate images.^[^ ^18^ ^]^ An open‐source Python script, numpy‐stl, was customized to generate 3D models in stl formats from the AI‐generated images for 3D printing.

## Conflict of Interest

The authors have filed patents for this research under No.63416931 and No.18818665.

## Author Contributions

M.G.L. supervised the research. M.G.L. and C.K.W.L. conceived the idea, and worked on conceptualization and methodology. Y.X., Y.H.C., W.Y.P., H.Z., and S.C. conducted the experiments. C.K.W.L. wrote the manuscript and worked on visualization. All authors discussed, revised, and finalized the manuscript.

## Supporting information



Supporting Information

Supplemental Video 1

Supplemental Video 2

## Data Availability

The data that support the findings of this study are available from the authors upon request.
