# Supplementary material for: Advanced 3D Food Printing with Simultaneous Cooking and Generative AI Design
Source: Adv Mater. 2025 Feb 26;37(13):2408282. doi: 10.1002/adma.202408282 (PMC11962674; doi:10.1002/adma.202408282)
Supplement: Supplementary file 1 — Supporting Information [file ADMA-37-2408282-s002.docx]

Supporting Information

**Advanced 3D Food Printing with Simultaneous Cooking and Generative AI Design**

*Connie Kong Wai Lee, Yang Xu, Qiaoyaxiao Yuan, Yee Him Chan,
Wing Yan Poon, Haosong Zhong, Siyu Chen, Mitch Guijun Li^*^*

**File name:** Supporting Information

**Description:** Supplementary Figures and Tables.

**File name:** Supplementary Video 1

**Description:** COMSOL simulation animation of the heat transfer process with a simulated heat flow is applied at 6.5 W onto the dough, distributed in a Gaussian beam with a radius of 4 mm.

**File name:** Supplementary Video 2

**Description:** The shape retention ability of starch-based dough during the printing process was recorded with camera and IR imaging at the 30-minute interval, utilizing the in-line LIG infrared heating method.

**Supplementary Figures**


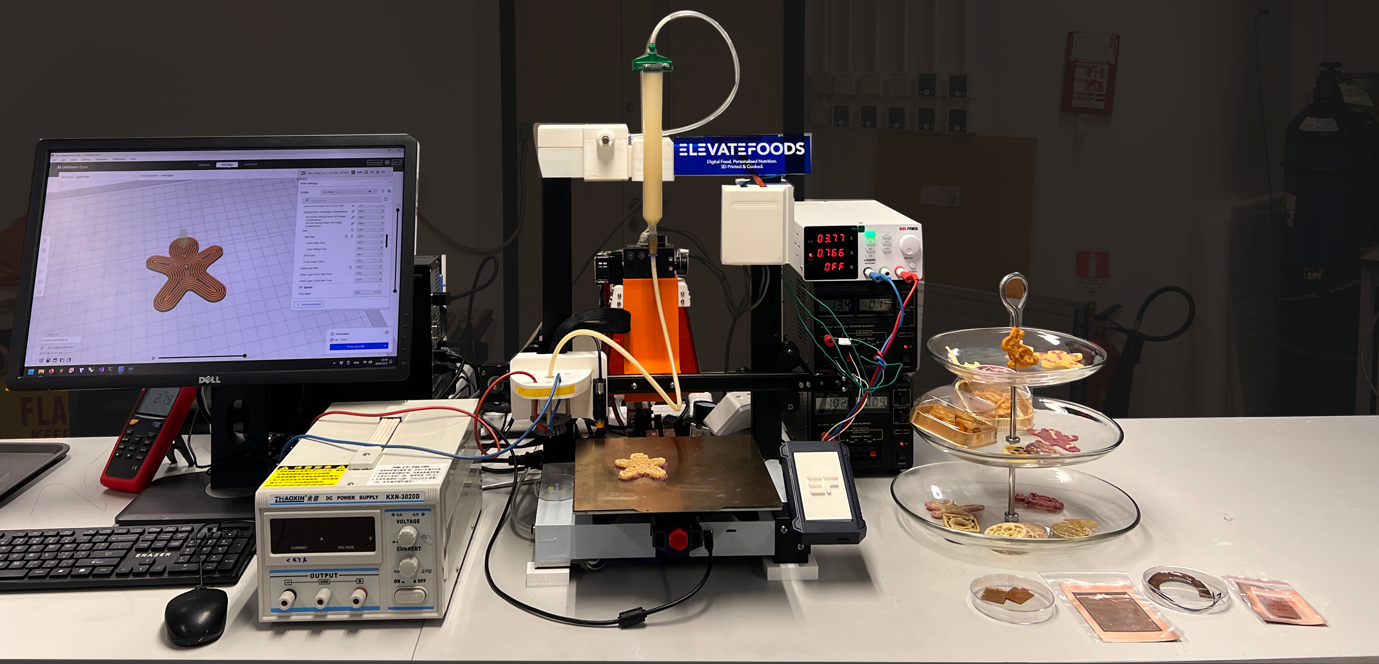


Figure S1. Working prototype of the integrative 3D food printer.


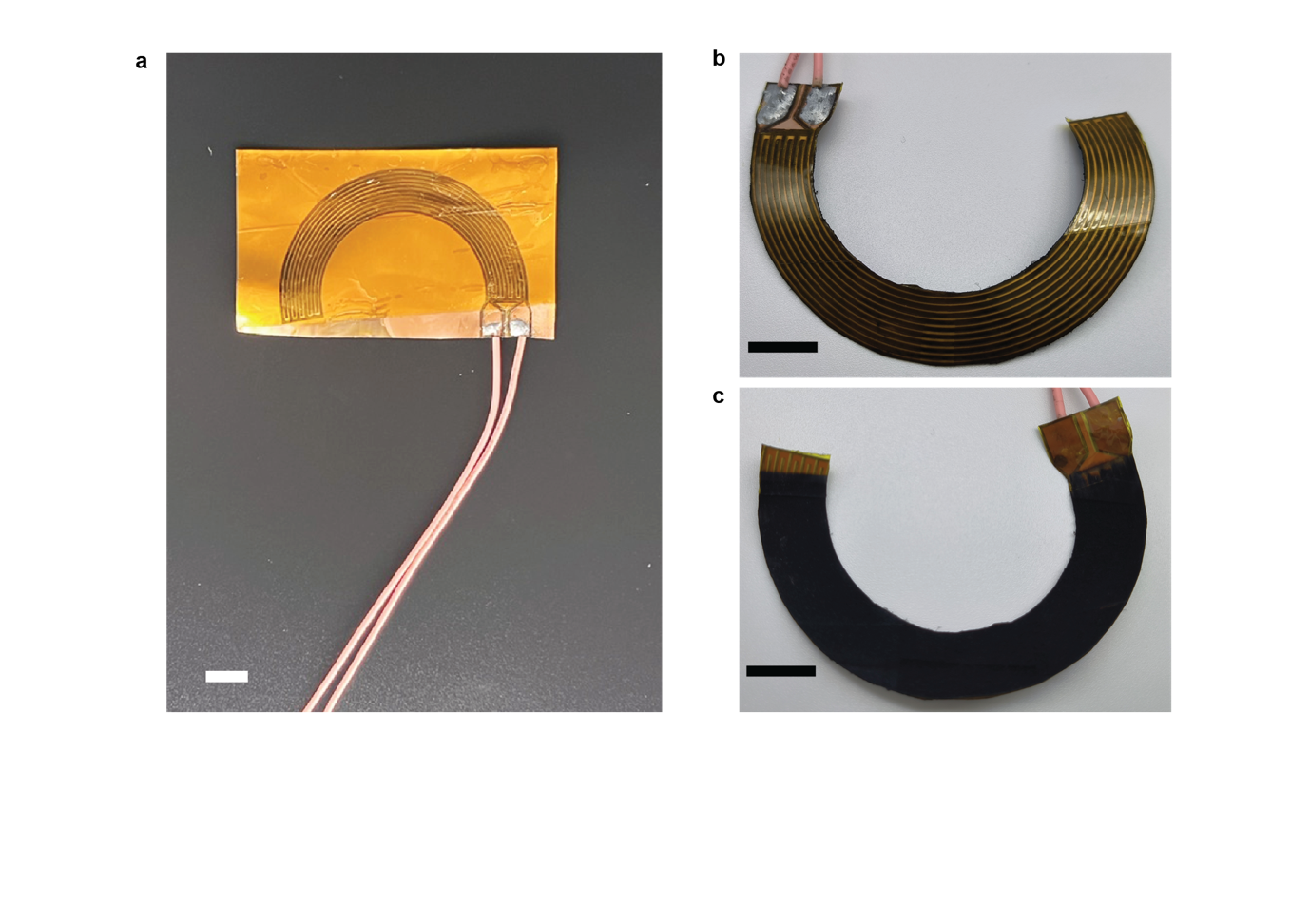


Figure S2. (a) Fabricated sample of the laser-scribed Cu pattern on a CuPI film before trimming. (b) Trimmed U-shape of the laser-scribed CuPI film. (c) Trimmed U-shape of the LIG-coated CuPI film. Scale bars (a-c) are 1 cm.


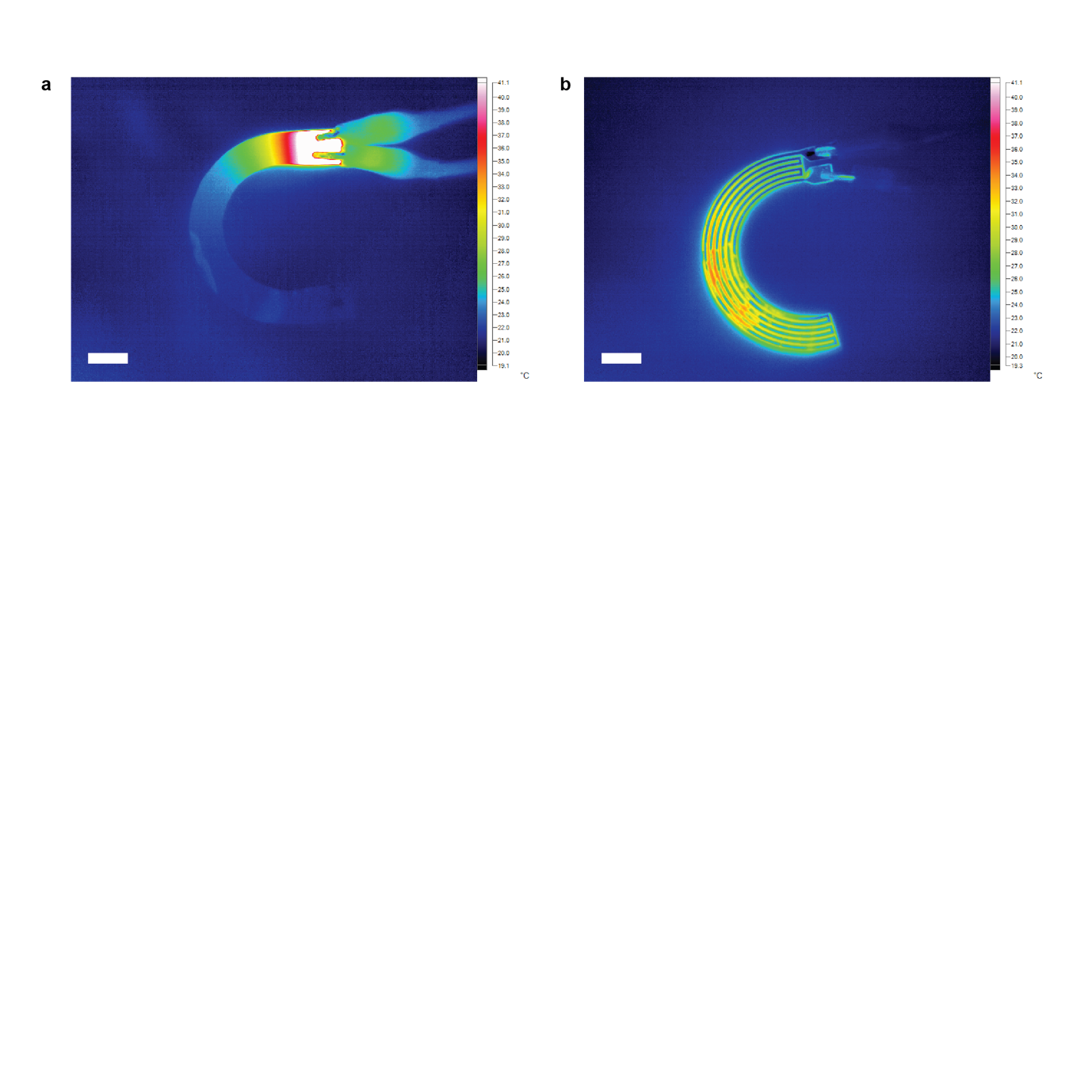


Figure S3. (a) IR camera image of non-patterned CU heating film. (b) IR camera image of patterned LIG heating film. Scale bars (a-b) are 1 cm.


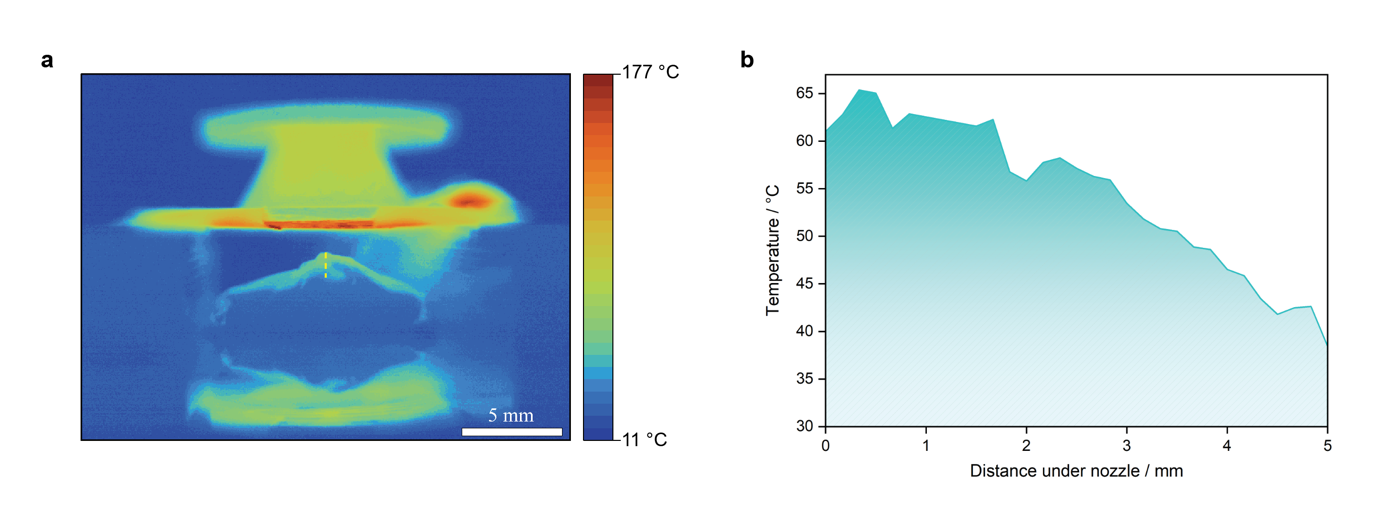


Figure S4. IR Temperature profiles of the printed dough with flat-surface LIG heating film. (a) IR camera image of the printed dough. (b) Temperature of the printed dough during the printing process.


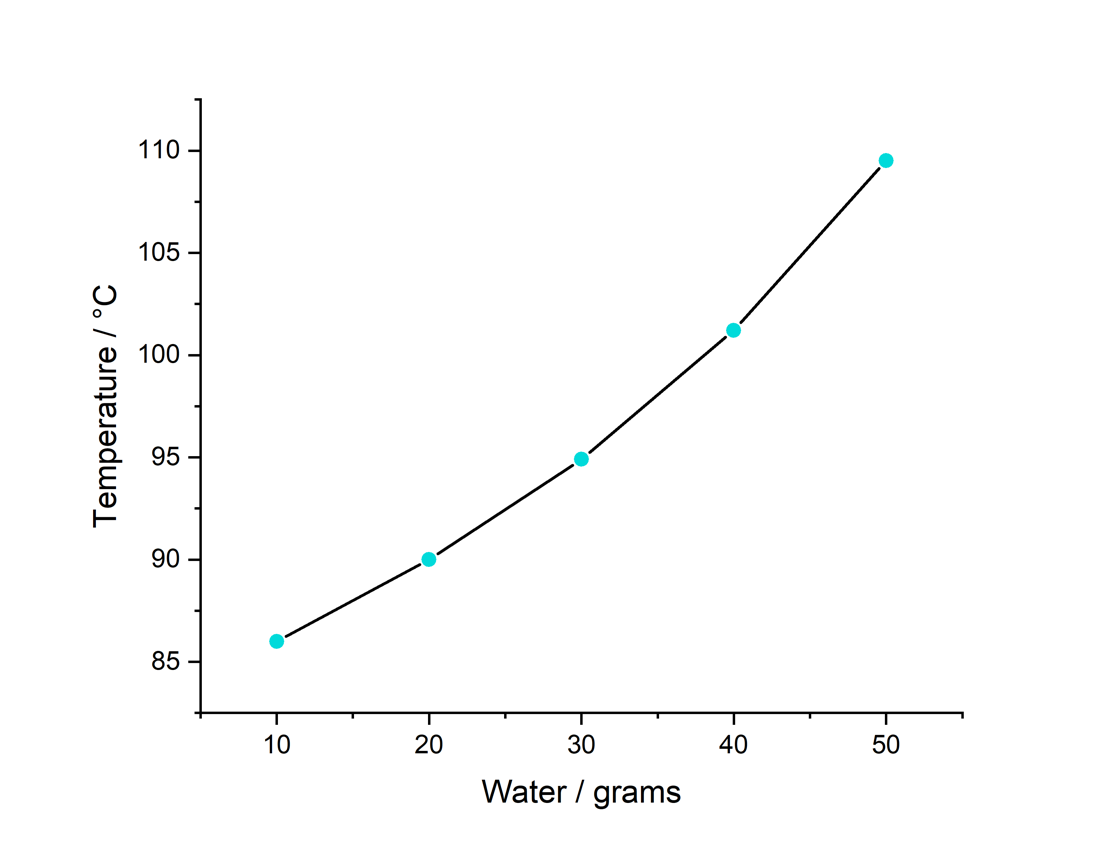


Figure S5. Maximum temperature at different water concentrations based on COMSOL simulations.


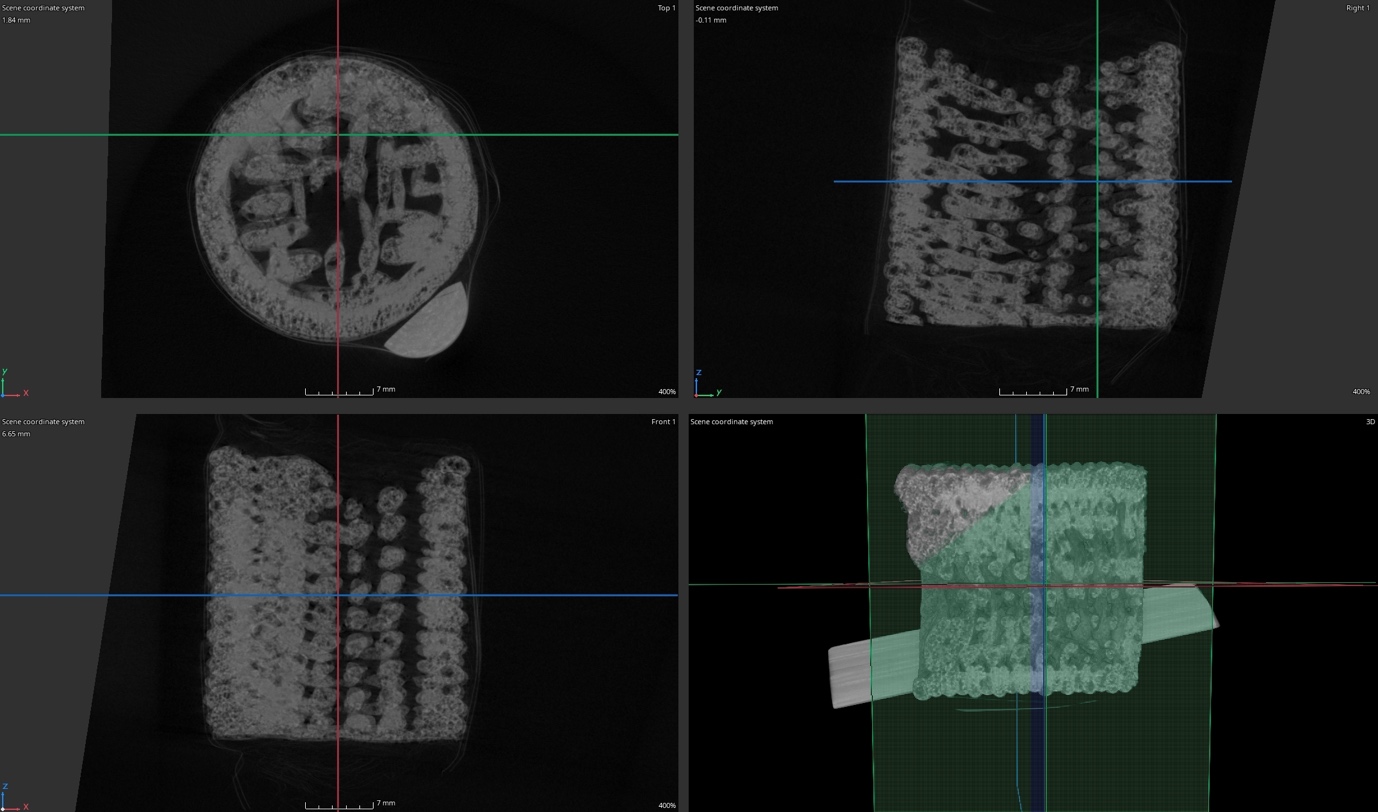


Figure S6. Micro-CT scanned 3D model of the IR cooked dough.


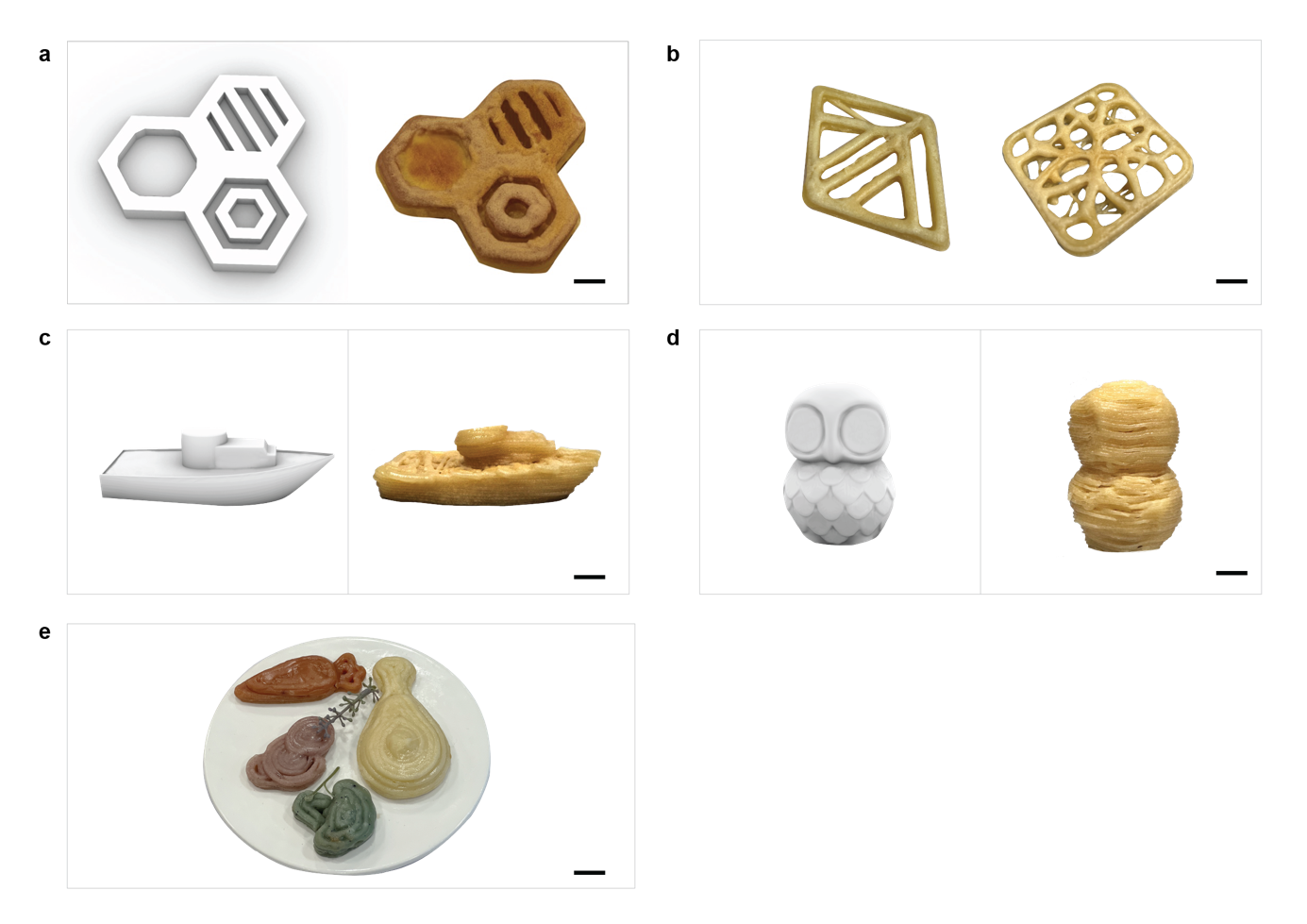


Figure S7. Printed food samples with complex shapes. (a-b) Cooke dough shapes with various perforated patterns. (c) 3D toy boat model cookie dough. (d) Standing owl model cookie dough. (e) Printed food samples with vegetable puree and chicken puree.


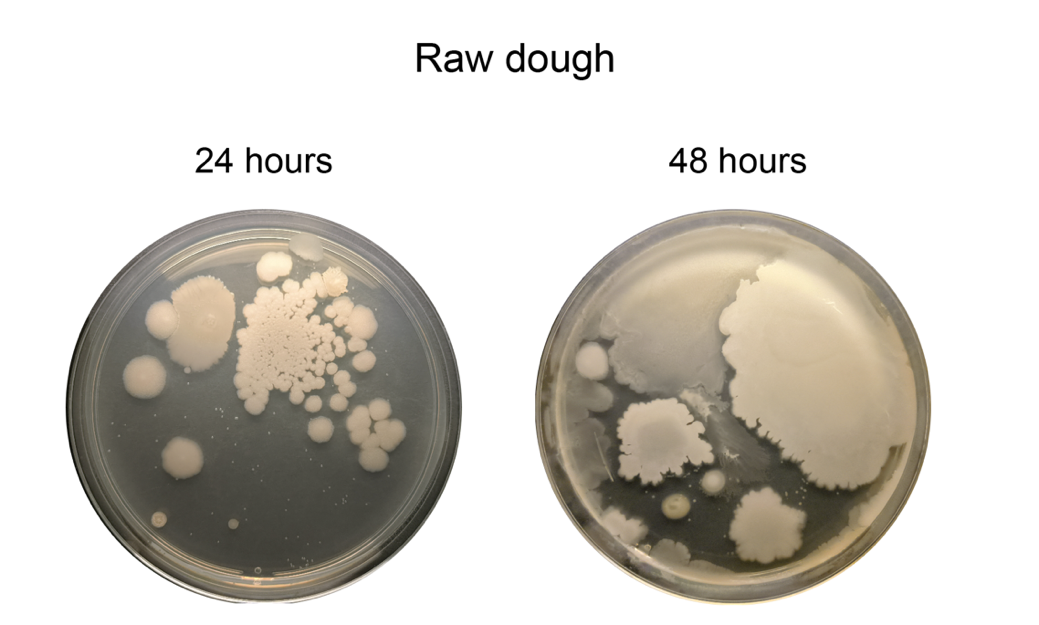


Figure S8. Bacteria colony counting image of raw dough at 24 and 48 hour post cultivation.


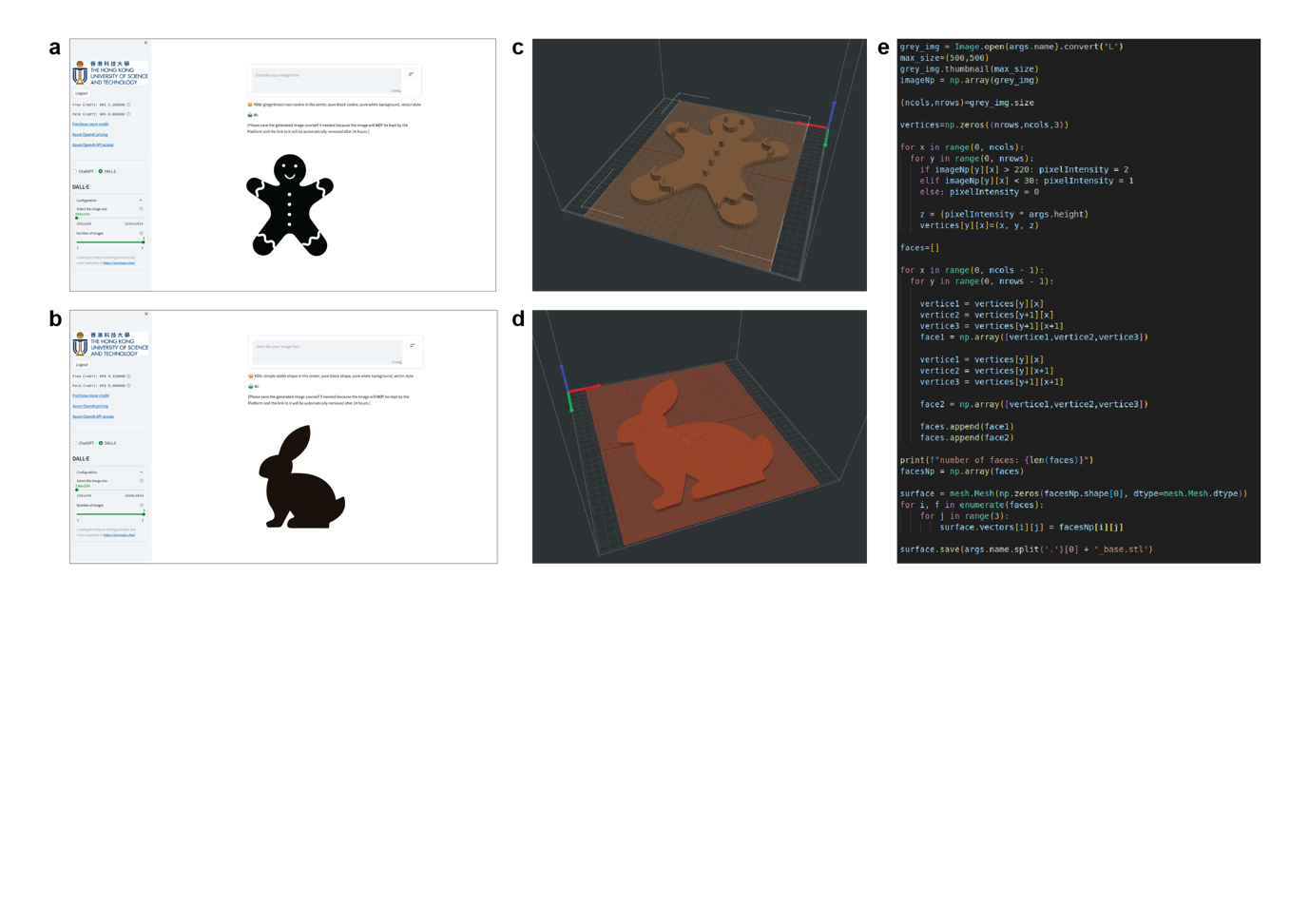


Figure S9. (a) Generative AI result of creating a gingerbread man cookie, and (b) a rabbit shape with DALL-E text-to-image model. (c) A STL file of the gingerbread man cookie image and (d) rabbit shape for printing, generated from Python script. (e) Coding of the Python script to generate double layers of extrusion based on the color codes of the imported images.


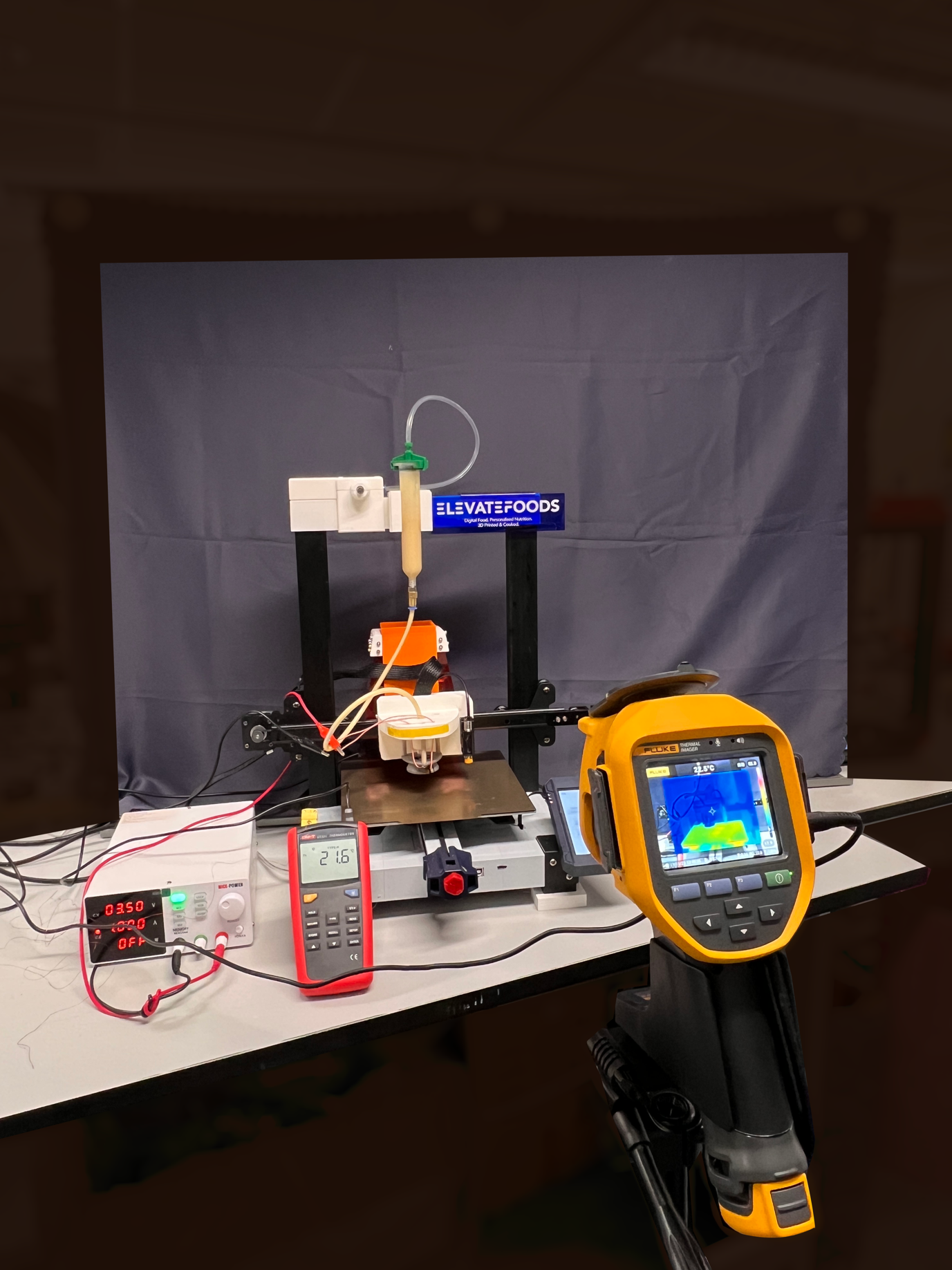


Figure S10. Experiment setup during the printing process with infrared imaging and K-type thermocouples to obtain the temperature profiles of the printed dough.


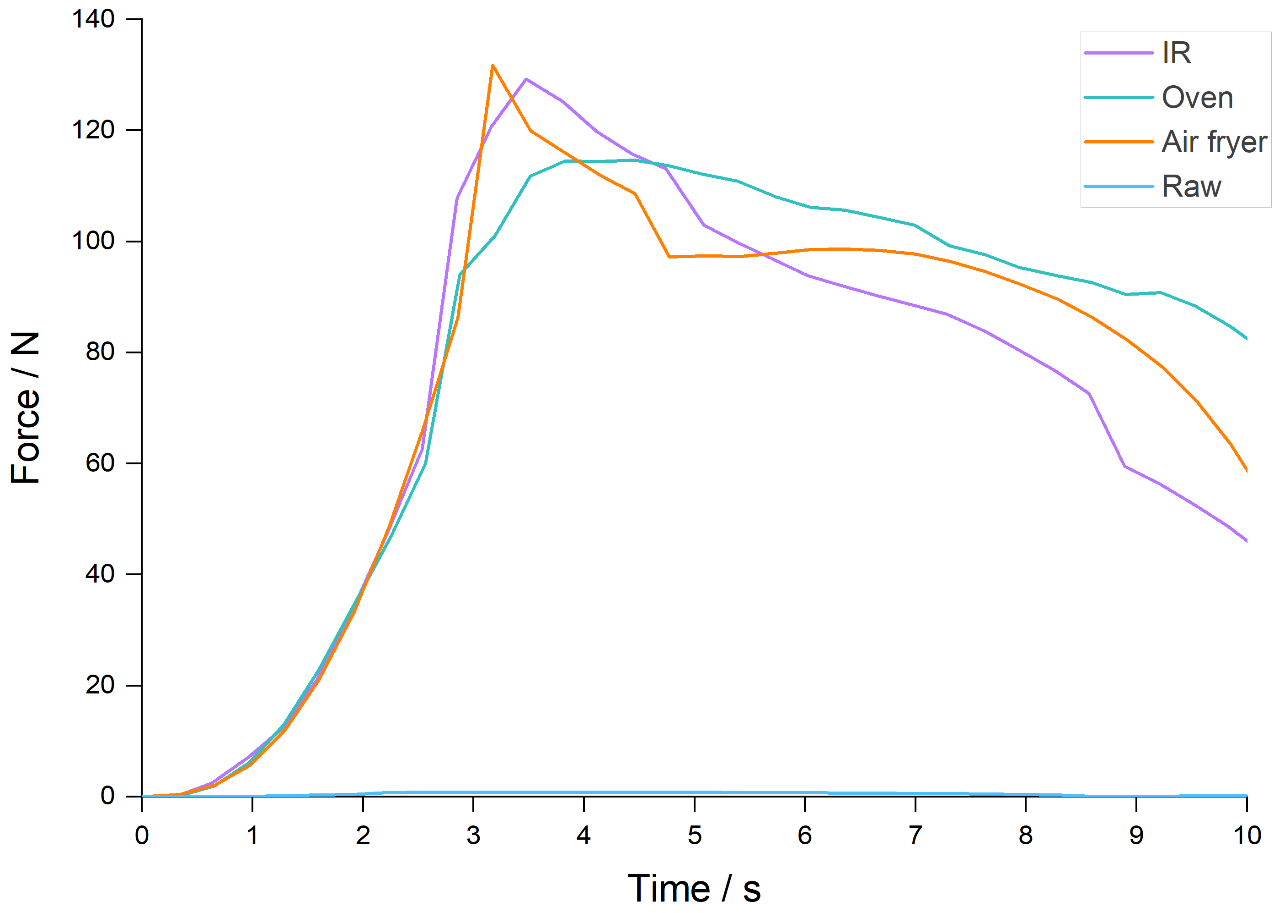


Figure S11. Force-loading force of IR-cooked, oven-baked, and air-fried cookie dough samples using a pin penetration test.


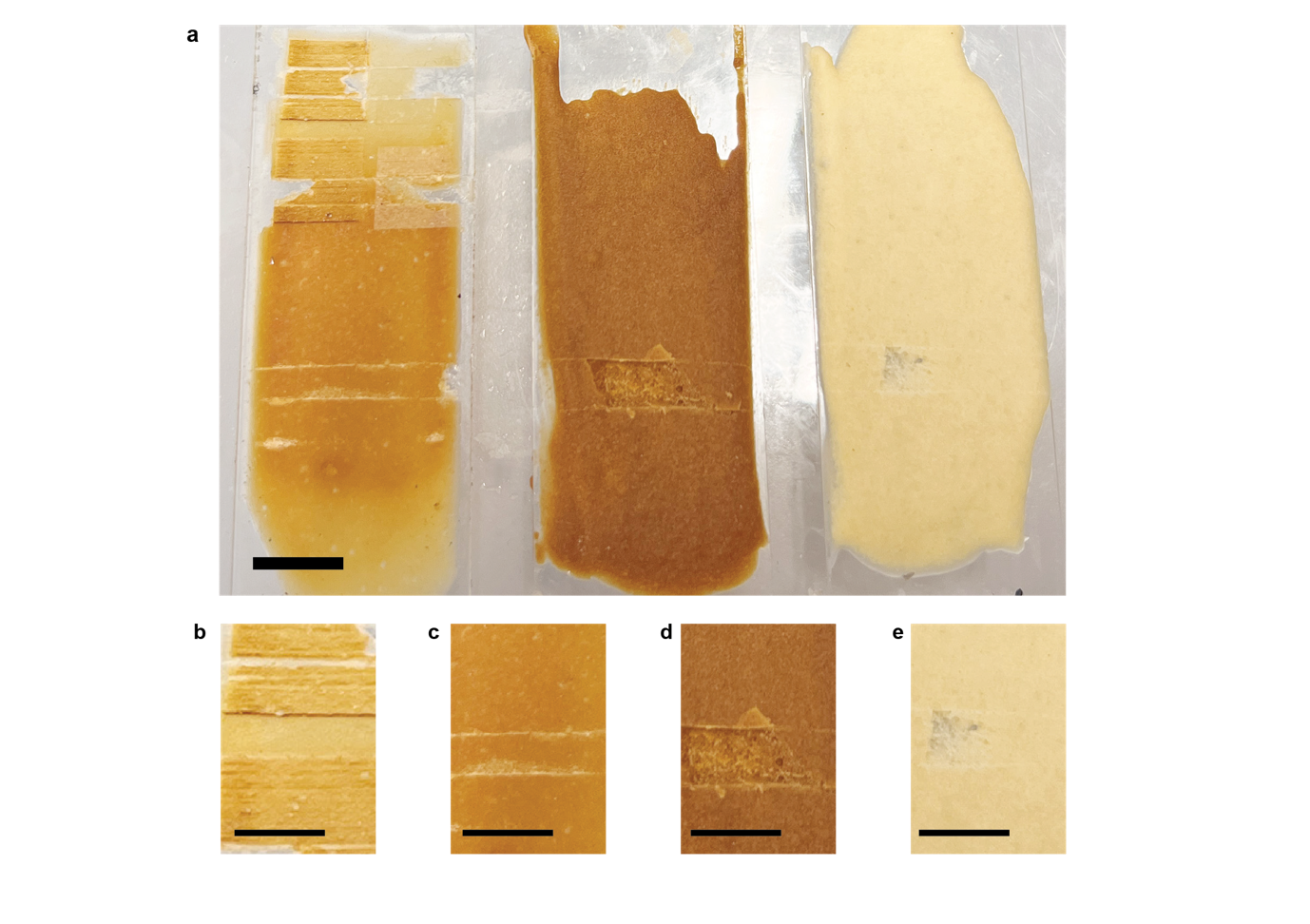


Figure S12. (a) Starch-based food dough samples with different cooking methods for SEM analysis (b) Photograph of the laser-cooked dough sample. (c) Photograph of the IR cooked dough sample. (d) Photograph of the air-fried dough sample. (e) Photograph of the oven-baked dough sample. Scale bars (a-e) are 1 cm.

**Supplementary Tables**

| Ingredients | Soft wheat flour | Sugar syrup | Vegetable oil | Water |
| --- | --- | --- | --- | --- |
| Quantity (g) | 78 | 60 | 24 | 25 |

Table S1. Recipe of the starch-based cookie dough for experiments.

| Nozzle size | 1 mm |
| --- | --- |
| Print speed | 13 mm/s |
| Travel speed | 80 mm/s |
| Air pressure | 2.0 PSI |
| Printing temperature | Ambient |
| Print bed material | Polyetherimide coating |
| Print bed temperature | 100 °C |
| Syringe volume | 50 cc / 70 cc |
| Layer height | 0.7 mm |
| Infill density | 80-90% |
| Infill pattern | Rectilinear |

Table S2. 3D food printer and slicing settings.
